# Tumor Microenvironment Immunosuppression: A Roadblock to CAR T-Cell Advancement in Solid Tumors

**DOI:** 10.3390/cells11223626

**Published:** 2022-11-16

**Authors:** Abigail Johnson, Michelle Townsend, Kim O’Neill

**Affiliations:** Department of Microbiology and Molecular Biology, Brigham Young University, Provo, UT 84602, USA

**Keywords:** tumor microenvironment, TME, chimeric antigen receptor, CAR T cell, cancer, immunotherapy, solid tumor, immunosuppression, CAR T-cell exhaustion

## Abstract

Chimeric antigen receptor (CAR) T cells are an exciting advancement in cancer immunotherapy, with striking success in hematological cancers. However, in solid tumors, the unique immunosuppressive elements of the tumor microenvironment (TME) contribute to the failure of CAR T cells. This review discusses the cell populations, cytokine/chemokine profile, and metabolic immunosuppressive elements of the TME. This immunosuppressive TME causes CAR T-cell exhaustion and influences failure of CAR T cells to successfully infiltrate solid tumors. Recent advances in CAR T-cell development, which seek to overcome aspects of the TME immunosuppression, are also reviewed. Novel discoveries overcoming immunosuppressive limitations of the TME may lead to the success of CAR T cells in solid tumors.

## 1. Introduction

### 1.1. CAR T Cells

**Figure 1:**
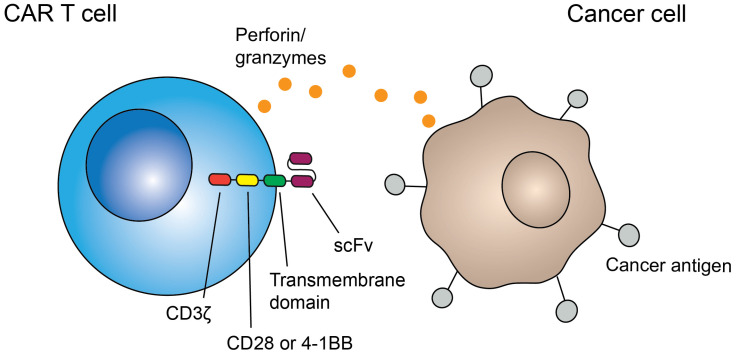
CAR T-cell structure and effector mechanism. CAR T cells are composed of a CD8+ T cell with a CAR construct, which is composed of scFv, transmembrane, and activation domains. CAR T cells bind to their specific antigen on cancer cells, activating cytotoxic function (directional release of perforin/granzymes or Fas/FasL interaction), killing the cancer cell.

Cancer immunotherapy is a growing and important field, shaping the future of cancer treatment. One category of immunotherapy seeks to selectively kill cancer cells through specific targeting of tumor-associated or tumor-specific antigens using chimeric antigen receptors (CARs). CARs are a combination of the variable binding portion of an antibody with the signaling and costimulatory domains of a T-cell receptor. The variable binding portion is typically a single-chain variable fragment (scFv), comprised of a portion of the heavy and light chain of an antibody (Figure 1). In a CAR, an scFv is connected to a transmembrane domain by a hinge region. It also includes intracellular signaling domains: the primary signaling domain, CD3ζ, and costimulatory domains, commonly CD28 and 4-1BB [1]. This CAR construct allows CAR T cells to be activated with a single binding event, eliminating the need for coreceptor interaction, which is necessary for activation of normal T cells. Upon binding, the CD8+ T-cell cytotoxic capabilities, including directional release of perforin and granzymes and Fas/FasL interactions, are then activated, killing the target cell [2,3].

CAR T-cell therapies were first FDA approved in 2017 for treatment of pediatric and young adult acute lymphoblastic leukemia (ALL) and later other B cell malignancies. Tisagenlecleucel (Kymriah^®^), developed by Novartis, was the first to be approved, with Axicabtagene ciloleucel (Yescarta^®^) developed by Kite Pharma following several months later [4]. After this initial success, research applying CAR T cells to many other cancers increased dramatically in the immunotherapy community. Currently, there are six FDA approved CAR T-cell therapies, all for hematological cancers (Abecma^®^, Breyanzi^®^, Carvykti^®^, Kymriah^®^, Tecartus^®^, and Yescarta^®^), with an overall response rate above 80% for B cell malignancies [5,6,7]. The most recently approved CAR T-cell therapy, ciltacabtagene autoleucel, has two binding domains to B-cell maturation antigen (BCMA), a B-cell marker that becomes over-expressed in multiple myeloma [8]. The success of this treatment was reported at 97% overall response rate and 67% complete response rate [8]. CAR T-cell therapy is quickly becoming a more common treatment option for patients with relapsed and refractory hematological cancers [9]. CAR T-cell research continues with over 500 clinical trials currently [5]. These CAR T cells have many modifications to alter their mechanisms and apply them to a variety of cancer types.

Despite the impressive success of CAR T-cell therapy in blood cancers and the plethora of research applying the principle to other cancers, CAR T cells have yet to be as successful in solid tumors. Solid tumors have an influential tumor microenvironment (TME), which presents obstacles to CAR T-cell homing to the tumor, activation, and longevity [10]. The characteristics of the TME, its mechanisms of immunosuppression, and how that affects CAR T-cell efficacy will be discussed in this review article.

### 1.2. Tumor Microenvironment

**Figure 2:**
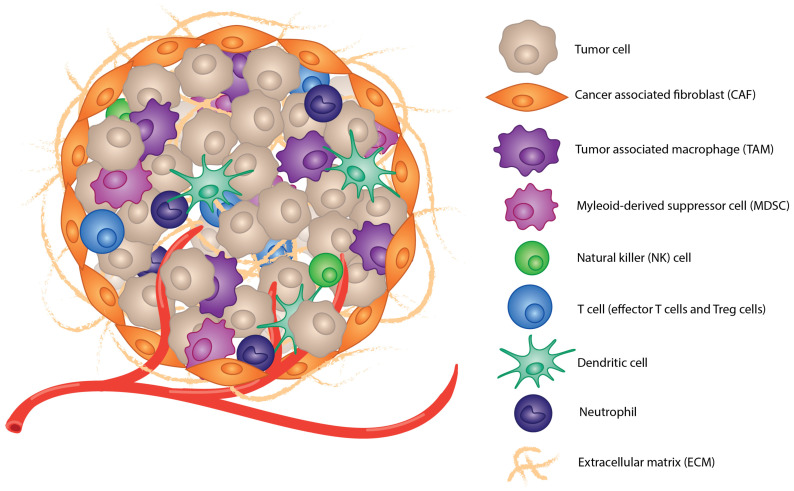
The heterogenous tumor environment. Tumors are comprised of a variety of cell types, as shown above. The ECM and CAF form a barrier that separates the tumor from the surrounding tissue. The vasculature of the TME often is insufficient for the constant proliferation and tumor growth.

The heterogenous components of the TME include a variety of cells, matrix proteins, and secreted factors. Cells in the TME include cancer cells, cancer-associated fibroblasts (CAFs), and immune cells, including tumor-associated macrophages (TAMs), natural killer (NK) cells, myeloid progenitor cells, myeloid-derived tumor suppressor cells (MDSCs), effector and regulatory T cells, dendritic cells (DCs), and neutrophils (Figure 2). Other cell types are present depending on the tumor location and cancer type. There is also an extracellular matrix (ECM) comprised of stromal cells, fibrous proteins, glycoproteins, proteoglycans, and polysaccharides, which provides structure to the tumor and gives some separation between other tissue [11]. The ECM also affects cell differentiation, proliferation, and metastasis [12,13]. Secreted factors, including cytokines, chemokines, and other proteins, are secreted by cancer and immune cells into the TME as well [14]. These secreted factors affect the TME in a variety of ways, including trafficking and polarization of immune cells and their activation or repression, cell growth and proliferation, and the inflammatory state of the TME [15].

Another physical characteristic of the TME is its irregular vasculature. Angiogenesis is considered a hallmark of cancer, and as tumors develop, a hypoxic state due to increased tumor mass without accompanying nutrient delivery triggers an “on switch” for angiogenesis [16]. Angiogenesis initiation is also influenced by secreted factors from surrounding cancer and immune cells and is often chronically activated in tumors [17]. The blood vessels produced by chronic activation of angiogenesis are commonly irregular, leading to areas of high and low circulation, excessive capillary branching, and blood vessel leakage, which can greatly affect cell populations and metastasis [18].

The players and ecosystem of the TME greatly influence the immunosuppression crucial to tumor growth and metastasis. This immunosuppression not only plays a role in the development of the tumor but also affects immunotherapy success. Though this discussion only scratches the surface of the complexities of the TME, the mechanisms of immunosuppression in the TME and its effect on CAR T-cell therapy will be further discussed.

## 2. Mechanisms for Immunosuppression

### 2.1. Immunosuppressive Cell Populations

As previously mentioned, there are many different immune cell populations present in the TME. Some are tumor-antagonizing, while many are tumor-promoting [19]. Tumor-antagonizing immune cells recognize the cancer cells as damaged, which stimulates an anti-tumor response.

Among these anti-tumor immune cell populations are CD8+ T cells, which have cytotoxic abilities to kill cancer cells when activated and are considered the key effector cell against cancer [20]. CD4+ T cells provide help and stimulation to CD8+ T cells and orchestrate the immune response through release of proinflammatory cytokines and chemokines [21]. NK cells are important for immunosurveillance of tumors, and they also have cytotoxic abilities similar to CD8+ T cells [22]. DCs are professional antigen-presenting cells (APCs) and mainly function in the TME to present antigen and give costimulation to T cells [23]. Macrophages are plastic cells and can be polarized to an M1-like or M2-like phenotype. M1 macrophages are pro-inflammatory macrophages that have anti-tumor function through phagocytosis of tumor cells and production of proinflammatory cytokines and reactive oxygen species [24]. Finally, neutrophils are important for releasing proinflammatory cytokines and contributing to a cytotoxic effect against cancer cells through phagocytosis, release of neutrophil extracellular traps, and antibody-dependent cellular-cytotoxicity (ADCC) [25].

In an ideal world, these tumor-antagonizing immune cells function to eliminate cancer cells as tumors form and prevent progression and metastasis [19]. However, in many cases, the variety of immunosuppressive mechanisms employed by the tumor—from metabolic changes to angiogenesis to immunosuppressive cytokine release—reduce the efficacy of anti-tumor immune cells and even turn them away from the TME [26]. The TME is catered to support pro-tumor immune cells, which assist in the suppression of an anti-tumor immune response.

TAMs are an influential pro-tumor immune cell population and comprise up to 50% of the tumor mass in some cancers [27]. Macrophages can polarize to become M1-like or M2-like in response to their environment. The TME both polarizes macrophages in the tumor to become M2 and recruits other M2 macrophages to the tumor [24,28]. M2-polarized macrophages induce immunosuppression, angiogenesis, tissue healing, and growth and downregulate inflammatory or M1 functions [29]. These characteristics of M2 macrophages that TAMs often have support tumor growth and metastasis. TAMs also tend to accumulate in necrotic or low-oxygen areas of tumors [30]. This accumulation plays a role in the pro-angiogenic effect of TAMs. TAMs release growth factors, including VEGF, PDGF, and TGF-β, as well as the angiogenic factor thymidine phosphorylase and other angiogenesis-modulating enzymes [27,31,32,33]. This directly influences the irregular angiogenesis, which is characteristic of the TME. Growth factors released by M2 macrophages also promote uncontrolled growth of cancer cells and increase tumor progression [34]. The cytokine profile released by TAMs (discussed in greater detail in the following section) interacts with other immune cells, preventing activation and recruitment of effector T cells, inducing Treg differentiation, and overall suppression of the inflammatory immune response. Higher levels of M2 macrophages are correlated with a poor prognosis in cancer patients [35]. These attributes demonstrate that TAMs greatly influence the immunosuppressive state of the TME and are highly significant in cancer progression.

MDSCs are immature myeloid cells that take one of two forms: (1) mononuclear MDSCs (M-MDSCs) which are quite similar and can later become TAMs, or (2) polymorphonuclear MDSCs (PMN-MDSCs), which phenotypically resemble neutrophils [36]. MDSCs affect the TME in a variety of ways similar to TAMs; however, their most prominent feature is their immunosuppression, especially inhibiting T-cell function [37]. They are effective at killing and inducing T-cell anergy by expressing high levels of programmed death ligand-1 (PDL-1), release of immunosuppressive cytokines, and sequestering essential amino acids and nutrients [36]. One important metabolic immunosuppressive mechanism of MDSCs is their accumulation of cysteine, which is necessary for T-cell activation. The depleted amount of cystine in the TME due to MDSC sequestering prevents T-cell activation—both native T cells and CAR T cells [38,39]. In clinical trials of CAR T cells, lower levels of MDSCs present in the tumor were correlated with more success of the CAR T-cell treatment, demonstrating the inhibitory effect MDSCs have on CAR T cells in the TME [40].

Regulatory T cells (Tregs) are immunosuppressive T cells most often characterized by expression of CD4, CD25, and FoxP3 [41]. In the body, they play an important role in maintaining homeostasis in immune response and influencing peripheral tolerance, which allows them to play a key role in preventing autoimmunity [42,43]. In cancer, the role of Tregs is complicated, but in several cancers, especially solid tumor cancers, the Treg population is increased during tumor progression and is correlated with a poor prognosis [44,45]. The role of Tregs is two sided. Tregs play a role in preventing chronic inflammation, which may be favorable in tumor prevention because, in some cancers, the chronic inflammatory state can induce mutations in cancer cells leading to tumor progression [46]. However, Tregs are also key players in tumor escape and immunosuppression by inhibiting cytotoxic T-cell elimination of cancer cells and through release of immunosuppressive cytokines (discussed further in the following section) [47].

CAFs contribute to a significant percentage of tumor mass [34]. Fibroblasts in normal tissue are a small proportion of cells that contribute to formation of connective tissue [48]. In tumors, CAFs play several roles but are primarily responsible for extracellular matrix regulation, which in turn affects tumor growth, immune invasion, and metastasis [49]. They are a heterogenous population of cells, which also release secreted factors and chemokines to affect the metabolism, angiogenesis, and immunosuppression of the TME [50,51]. Perhaps one of their most crucial immunosuppressive roles is as a physical barrier preventing immune cell infiltration. CAFs form a “shell” of sorts around the tumor, which is especially effective at preventing T-cell infiltration. A recent study confirms that CD8+ T-cell infiltration in breast cancer tumors is directly correlated with the strength of the CAF barrier, due not only to physical barrier but also due to secreted factors that affect the immunosuppressive effects of the TME [52].

### 2.2. Secreted Factors

Secreted factors, including cytokines and chemokines, are an essential part of immunosuppression in tumors, as they are a signaling mechanism between cells, especially immune cells. The most relevant cytokines to the TME are controlled by the transcription factors NF-κB and STAT3 [53]. NF-κB is important for the formation of inflammatory sites inside the tumor by inducing gene expression of inflammatory cytokines, most notably TNF-α, IL-1, and IL-6 [54]. STAT3 also induces gene expression of inflammatory cytokines and type-1 interferons, though arguably, its most important role is in expression of IL-6 [55]. The interplay between NF-κB and STAT3 controls a majority of the inflammatory cytokine production in the TME, which alters the immune balance there, recruiting immune cell populations such as TAMs and MDSCs [56].

Pro-tumor immune cells produce anti-inflammatory and immunosuppressive cytokines, which can reduce effector cell function and allow for tumor escape. Notable among these are IL-10 and TGF-β produced by TAMs, MDSCs, and Tregs [57]. IL-10 is important for helper T cells to perform immune surveillance and allow immunosuppressive function and is commonly produced by Tregs [58]. TGF-β is known to be involved in tumor progression, though members of this cytokine family induce a variety of effects on differentiation, metastasis, and tumor invasiveness [59]. Other cytokines/growth factors allow for direct recognition of cancer cells through innate immunity. For example, PDGF-D is secreted by tumor cells and is the ligand for NK cell-mediated recognition of tumor cells [60]. Immunosuppressive and inflammatory cytokines work together and against each other to affect the complex interplay of signaling and directing immune cell function, promoting cancer proliferation, and altering the TME (Table 1 [53]).

### 2.3. Metabolic Influences on Immunosuppression

The TME is characterized by an abnormal metabolic profile, which can be highly immunosuppressive. Some of these metabolic differences include insufficient nutrient and oxygen levels and accumulation of metabolic waste.

Cancer cells have a high demand for virtually every nutrient required for growth and proliferation, which often starves surrounding and invading immune cells of nutrients they need. Notably, glucose and glutamine are shown to be especially needed by cancer cells [61,62]. Cancer cells perform glycolysis for a majority of their energy, even in the presence of enough oxygen, in a phenomenon called the Warburg effect [63,64]. This phenomenon was first observed in the 1920s by Otto Warburg, when he and his colleagues observed the dramatically increased amount of glucose being consumed by tumor cells compared to surrounding tissue [65,66]. Glutamine’s necessary role in the synthesis of nucleotides and amino acids leads to significant use of glutamine in cancer cells as well [67]. Low levels of glucose in the TME can cause immune cells to be activated less and die at greater rates [64]. For example, naïve CD4+ and CD8+ T cells shift to upregulate glycolysis and glutaminolysis when they become activated. However, when glucose and glutamine levels are low in the TME, T cells do not activate as strongly, and Treg differentiation increases as well [68,69]. Other immune cells also require glucose and glutamine for survival and activation, and competition with cancer cells for these limited resources can cause most immune cells to be suppressed in the TME [68]. Cancer cells are also prone to rapid metabolite adaptation, where they can adjust to the changing metabolite concentrations in the TME, allowing them to not only survive but also outcompete other cells in the area [70].

Insufficient oxygen level, or hypoxia, is common in the TME because extensive proliferation is unsupported by proper blood vessel formation, which affects the function of all cells in the TME, including immune cells [71]. Hypoxia reduces activation of effector cells, including CD8+ T cells and NK cells, similarly to insufficient nutrient levels, and can cause their death and reduced cytokine production [72,73]. The decreased oxygen levels in the TME also promote suppressor immune populations, including Treg and M2 macrophages [64,74].

The Warburg effect also affects the acidity of the TME because excessive production of lactic acid, a product of glycolysis, lowers the pH [75]. The acidity of the TME negatively affects immune cell function, resulting in immunosuppression. High levels of lactic acid were shown to induce expression of M2 polarizing genes in macrophages, including *Vegf* and *Arg1*, and they were also able to show that this expression was important to tumor growth in mice [76]. Lactate was also shown to inhibit nuclear factor of activated T cells (NFAT), which is a key activation transcription factor in T cells and NK cells [68]. Not only did high lactate concentration correlate with lower activation markers, but it also was positively correlated with T and NK cell apoptosis and decreased cytokine production [77]. Lactate dehydrogenase also interacts with FoxP3, an important transcription factor for Treg differentiation, allowing Treg cells to have a metabolic edge in surviving the TME [69].

## 3. Effects of the Immunosuppressive TME on CAR T Cells

**Figure 3:**
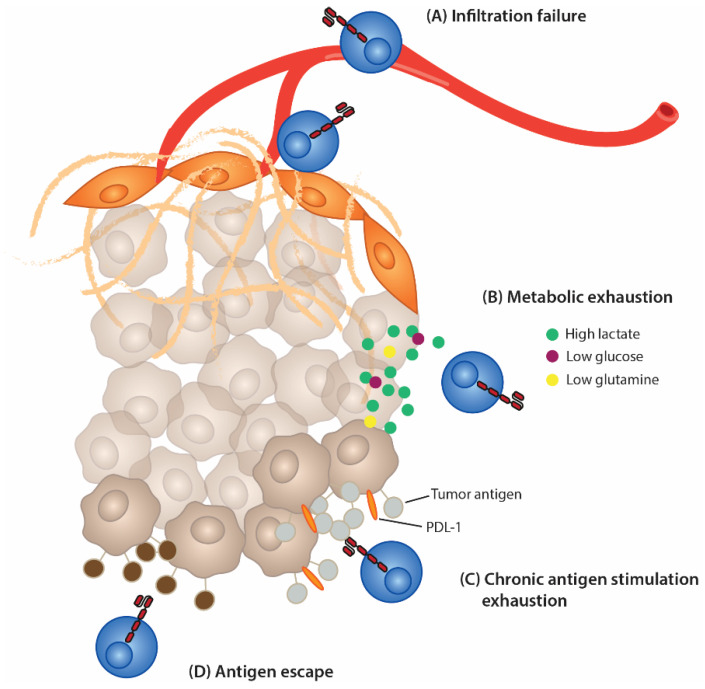
Immunosuppressive effects on CAR T cells. (**A**) CAR T cells can fail to infiltrate solid tumors due to lack of blood vessel penetration into the tumor, the barrier surrounding the tumor comprised of the ECM and CAFs, and down-regulation of extravasation mediators needed to leave the blood vessels. (**B**) Metabolic-induced exhaustion caused by increased glycolysis of cancer cells results in low nutrient levels and high waste levels. This metabolic environment impairs T-cell activation and cytotoxic function. (**C**) Chronic antigen stimulation from abundant cancer cells in the TME can lead to anergy and exhaustion of CAR T cells. (**D**) Heterogenous cancer cell populations with varying antigens and selective pressure can lead to antigen escape, where cancer cells will evolve not to express the antigen the CAR T cell binds to.

CAR T cells are considered “living drugs” because they activate, proliferate, and expand depending on what they are presented with and the enviornment they are in. This means that the immunosuppressive elements of the TME previously discussed can greatly affect the efficacy of CAR T cells. The TME can limit homing to tumor sites, limit infiltration into the tumors, induce exhaustion, and even impact acquired resistance to CAR T-cell therapy (Figure 3).

### 3.1. Homing and Solid Tumor Infiltration Difficulties

CAR T-cell therapy is significantly more effective in hematological cancers compared with solid tumor cancers primarily because of “accessibility” [78]. Patient data and in vivo imaging show that after treatment, CAR T cells are often present in high levels in the blood and lymph without successfully infiltrating the tumor [79,80]. Several elements of the TME contribute to the difficulty CAR T cells have in homing to and infiltrating solid tumors. CAR T cells are typically administered intravenously, though injection into the tumor is another option currently being studied. Thus, for CAR T cells to home to the tumor, they must travel through blood vessels. The insufficient vasculature of the TME impairs arrival of CAR T cells in the tumor because of lack of access [78]. This lack of access is also added upon by the “shell” surrounding the tumor comprised of CAFs and ECM [81,82]. Prevention of T-cell accumulation around and in solid tumors has also been shown to be correlated with high levels of MDSCs and TAMs, likely through secretion of inhibitory chemokines [81]. T cells leave the blood vessel and enter tissue through a process called extravasation, which relies on integrins and selectins for T cells to pass through the endothelial cells of the blood vessels [83]. However, studies on non-CAR T cells show that the TME impairs T-cell extravasation through irregular vasculature caused by tumor-induced angiogenesis and by downregulating extravasation mediators (including L-selectins, LFA-1, Mac-1, ICAM-1, and TNF-α) [84,85]. TAMs have also been shown to impair the ability of T cells to infiltrate into tumors. When TAMs were eliminated from a tumor mouse model with PLX3397, an inhibitor of colony-stimulating factor-1 receptor, the CD8+ T-cell population increased in strong correlation to macrophage depletion [86]. These studies demonstrate how T-cell infiltration is inhibited in solid tumors, but it is logical to deduce that CAR T cells would interact similarly because they are simply T cells with modified receptors. Therefore, we conclude that immunosuppression caused by ineffective CAR T-cell infiltration into solid tumors plays a major role in CAR T-cell failure in tumors.

One method that has become a common practice in CAR T-cell testing in animal models and clinical trials is injection of CAR T cells into the tumor rather than intravenous delivery. Some improvement of CAR T-cell homing and infiltration in solid tumors has been seen, but it has not shown a complete fix of the problem [87,88].

### 3.2. CAR T-Cell Exhaustion

T-cell exhaustion is a phenomenon where T cells experience decreased effector function and a different transcriptional profile, including continuous upregulation of inhibitory receptors [89,90]. T-cell anergy can appear functionally similar to T-cell exhaustion, with similar results of unresponsiveness to antigen, and also is common in the TME. Anergy is believed to be caused by either robust inhibitory costimulatory signaling or weak positive costimulatory signaling when T cells bind to peptide/MHC complexes [90]. CAR T-cell exhaustion is mostly influenced by a lack of oxygen and nutrients in the TME. T cells are sensitive to the metabolic environment in which they find themselves, and studies show that T-cell efficacy is greatly influenced by the metabolic state of the TME [91]. Immune suppressive metabolic elements previously discussed—including hypoxia, nutrient competition, and acidity—cause epigenetic changes to T cells in vivo, which alters their transcriptome and, thus, function to have significantly impaired anti-tumor abilities [91]. These same immunosuppressive metabolic elements are likely to alter the efficacy of CAR T cells in solid tumors as well.

Tumors have dense concentrations of cancer cells, which in turn creates very high levels of antigen stimulation. CAR T cells thus experience chronic antigen activation, influencing development of exhaustion [92,93]. The mechanism of how repeated antigen stimulation induces an exhaustive or anergic state is not well understood, but recent studies show that inhibition of mitochondrial oxidative phosphorylation leads to epigenetic changes and decreased expression of effector genes [94]. These changes in mitochondrial oxidative phosphorylation also affect mitochondrial depolarization and induce T cells toward terminal exhaustion [93]. Continuous activation also pushes CAR T cells towards exhaustion through increased expression of PD-1, which not only increases chances of death through interaction with PDL-1 on cancer cells but also may alter metabolic reprogramming to push T cells towards an exhausted state, even without direct interaction with PDL-1 expressed on cancer cells [95,96,97,98].

Some data show that using less differentiated T cells in CAR T-cell development improves persistence in the TME through greater capacity for proliferation and generation of a long-term memory response with fewer signs of exhaustion [99].

### 3.3. Antigen Escape in CAR T-Cell Therapy

Limited efficacy of CAR T cells in solid tumors can put selective pressure on the surviving cancer cells to become resistant to CAR T-cell therapy. [100] This can happen through loss of antigen, where the cancer cells mutate to lose the antigen that the CAR T cell recognizes or proliferate from a cell without the antigen that existed from the time of treatment [101]. The mechanisms and incidence of antigen escape-mediated resistance has been extensively reviewed by Lemoine et al. [102]. Increased expression of PD1-L can also affect resistance by killing the CAR T cells in large quantities through overexpression of the ligand [103,104,105]. Mechanisms of resistance to CAR T cells are still under investigation, and we expect to understand this phenomenon better in the future.

## 4. CAR T-Cell Advancements and Co-Treatments to Overcome Immunosuppression Roadblocks

### 4.1. Immune Checkpoint Inhibitors

Checkpoint inhibitors are another cancer immunotherapy that have seen significant success in recent years. Monoclonal antibodies against PD-L1 or CTLA4 have been developed to block binding to PD1 on native immune cells or CAR T cells [106]. Combination therapy of checkpoint inhibitors with CAR T-cell therapy has been tested in clinical trials with promising results. Checkpoint inhibitor therapy has been tested to determine whether it is more effective administered at the time of CAR T-cell infusion or at a certain time point post-infusion. Though effective in many patients regardless of treatment timeline, the best time of treatment is unclear [107]. Although combination therapy was effective in some patients, resistance did occur, indicating that there are other factors at play that are affecting CAR T-cell inefficacy [102].

Modifications have also been made to CAR T cells allowing them to evade the problems associated with PD1/PD-L1 interaction in tumors. In one study, an armored CAR was developed with the ability to secrete anti-PD1 scFvs when activated [108]. In a mouse model of both hematologic and solid tumors, their armored CAR T cell exhibited equal or better tumoricidal function compared with separate co-treatment of an anti-PD1 antibody and CAR T cell. In this therapeutic approach, the anti-PD1 scFvs remained localized to the tumor, unlike the systemic dispersal when administered in the traditional intravenous way, which limits potentially harmful immunosuppressive effects in other parts of the body [108]. Another way to alter checkpoint inhibition in CAR T cells is to make them resistant to PD-L1. PD-L1-resistant CAR T cells have been engineered by knocking out the PD1 gene using CRISPR/Cas9 technology in T cells before transduction [109,110,111]. Another method of creating PD1-resistant CAR T cells involves engineering a chimeric PD1/CD28 receptor, which alters the response when extracellular PD1 is bound from an inhibitory signal to a positive CD28 costimulatory signal [112]. Both of these PD1-resistant CAR models have increased efficacy in mouse models, though they have yet to be tested in clinical trials.

### 4.2. CAR Variations

Many variations have been made to the structure or composition of CARs and CAR T cells to improve their efficacy in solid tumors, the extent of which is beyond this review (see cited reviews for extensive detail on many different CAR varieties and applications) [5,113,114,115]. These modifications range from multi-specific CARs which bind multiple antigens to logic gated CAR T cells to cytokine secreting CAR T cells and CARs engineered into other cell types besides T cells [5].

Cytokine-secreting CAR T cells are an important development in CAR T-cell construction, also known as 4th generation CAR T cells, armored CARs, or TRUCKs [116]. These CARs are engineered so that upon binding of the scFv and activation of the signaling domains, nuclear factor of activated T cells (NFAT, an important T-cell activation transcription factor) is activated, triggering production and release of a specific cytokine, depending on how it has been engineered [117]. Many cytokines have been tested for use in these CAR T cells, including IL-7, IL-12, IL-15, IL-18, IL-2, and combinations of them as well [116]. The specific release of cytokines when CAR T cells are activated helps to alter the immunosuppressive TME by affecting the immune cell populations present and CAR T-cell stimulation, to improve both CAR T-cell response and overall immune response against the tumor [118]. Many cytokine-secreting CAR T cells are currently being tested, and there is significant promise in the clinical application of this CAR variation (see other sources for more information on current trials) [116,119].

One CAR variation that shows special promise in solid tumors are CAR macrophages (CAR-Ms). The construct of a CAR remains the same in CAR-Ms, though different signaling and costimulatory domains are being experimented with, but the CAR is incorporated into a macrophage instead of a T cell [120]. Several innate features of macrophages lend them to CAR-M application in solid tumors [121]. Macrophages have increased homing abilities to find tumors because they upregulate CCR2, which binds CCL2 (a chemokine secreted by tumor cells) [122]. They also have important plasticity, where they can alter between M1- and M2-like phenotypes depending on their stimulation. Macrophages are also professional APCs and can phagocytose cancer cells and present their antigen to other immune cells, generating a more robust immune response. CAR-M research has shown that they often have increased infiltration ability into solid tumors, and they can reduce the immunosuppressive state in the tumor by influencing polarization of surrounding immune cells and changing the cytokine profile [121]. CAR-M therapy was shown to recruit T cells to the tumor and increase presentation of cancer antigen to these T cells [123]. Research into this application of CARs is still ongoing, but it shows promise in solving many of the problems CAR T cells have in solid tumors.

There are many developments in CAR T-cell therapy where the binding domain is altered from the normal scFv [5]. One of these variations that may prove influential in solid tumors is CAR T cells that bind and are activated by soluble factors rather than membrane-bound surface antigen on cancer cells. CAR T cells were recently modified to bind to various soluble antigens, including the CD19 ectodomain and TGF-β, with the potential to engineer receptors for various soluble ligands with relative ease [124,125]. The receptors for these CAR T cells are structurally different from a typical scFv, but receptor dimerization allows for a cytotoxic response to be initiated [124]. These CAR T cells influence the TME by binding secreted factors that induce activation. For example, TGF-β binding CAR T cells reprogram the response to this cytokine from an immunosuppressive response to an immune-stimulating response [124]. Another CAR T-cell altered binding domain is incorporation of an NK cell ligand, NKp44, the ligands for which are found almost exclusively on and secreted by tumor cells [126,127]. One of these ligands is PDGF-DD, previously discussed as an NK cell tumor-recognizing receptor. These NKp44 CAR T cells offer another option for more widely directly targeting tumor cells, which often have more heterogenous expression of surface antigen [127]. CAR T cells may also bind this ligand when secreted to influence the TME, as mentioned above. NKp44 CAR T cells show effective anti-tumor effects in a variety of pediatric tumors and in synovial sarcoma [126,128].

### 4.3. Oncolytic Viruses in Combination with CAR T Cells

One approach to overcoming immunosuppressive effects of the TME is to use oncolytic viruses as an adjuvant to induce the tumor to change from an immunosuppressive to inflammatory immune environment. Oncolytic viruses work through selective invasion and subsequent killing of tumor cells [129]. Antigen-presenting cells (APCs) are then recruited, activating the innate anti-tumor immune response [130]. These viruses cause immunogenic cell death (ICD) through release of PAMPs and DAMPs [131]. Sustained ICD causes activation of an immune response that alters the TME through increased infiltration of immune cells stimulated by the release of antigens from lysed tumor cells [132]. There is currently one oncolytic virus approved by the FDA (discussed further below), and there are over 400 clinical trials for varying oncolytic viruses which have achieved varying anti-tumor success both as monotherapies and in conjunction with other immunotherapies [133,134,135]. However, current discoveries suggest that oncolytic viruses as a monotherapy are not likely to be fully effective due to the heterogenicity of cancer and its complex, dynamic nature [129].

The only oncolytic virus currently approved by the FDA, talimogene laherparepvec (T-VEC), is derived from human herpes simplex virus 1 and engineered to preferentially infect melanoma cells [135,136]. It encodes a granulocyte macrophage colony-stimulating factor, which stimulates a direct anti-tumoral effect and a secondary activation of APCs, resulting in an innate and adaptive immune response. T-VEC was determined to be well tolerated and safe, with a built-in safety of still being susceptible to common anti-viral drugs, including acyclovir [137]. Clinical trials obtained around 27% overall response rates and were shown to reduce Treg and MDSC levels [138,139,140]. The effect of T-VEC on the TME suggests it has potential to stimulate the immune response in the tumor to allow CAR T cells to be more effective. Oncolytic viruses are currently being refined to improve their anti-tumor effect, especially in tandem with CAR T-cell therapies. These prospects are extensively discussed in a recent review by Mardi et al. [129].

One promising novel oncolytic adenovirus was engineered to be “armored” with the CD8+ T cell attracting chemokine CXCL1 and used in conjunction with CAR T-cell therapy [141]. The CXCL11 secretion was hypothesized to support CAR T-cell infiltration into the tumor and potentially increase infiltration of other lymphocytes expressing CXCR11. In an immunodeficient glioblastoma mouse model, the combined treatment of armored oncolytic adenovirus (oAd-CXCL11) with a B7H3 binding CAR T cell (B7H3.CAR-T) was shown to be significantly more effective at reducing tumor progression and even tumor elimination than either treatment alone. In a fully immunocompetent glioblastoma mouse model, the oAd-CXCL11 was combined with B7H3.CAR-T, and the combination was not only the most effective at reducing tumor progression and prolonging life of the mice but also increased infiltration of natural M1 macrophages, NK cells, and CD8+ T cells into the tumor, causing a shift in the TME profile. The combination of adenovirus-induced activation of the innate immune system and chemokine-induced lymphocyte infiltration proves a promising mechanism for altering the TME and increasing CAR T-cell infiltration and efficacy [141].

Oncolytic viruses, in conjunction with CAR T-cell therapy, provide a promising solution to several of the TME-associated challenges of CAR T cells. Generation of a natural immune response, as well as engineered cytokine and chemokine secretion, allow oncolytic viruses to improve CAR T-cell homing to tumors [129]. The alteration of the TME’s immune environment may also improve CAR T-cell longevity and efficacy. There are virtually limitless combinations of oncolytic viruses and CAR T cells that can be combined as a form of personalized medicine for cancer patients [129]. These logical reasons make this combination therapy a promising method for targeted tumor destruction and TME alteration.

### 4.4. Metabolic Modifications

Modifications can be made to the CAR T cells themselves to improve metabolic function and overcome immunosuppression due to the unique metabolic profile of the TME. One mechanism is to use CRISPR/Cas9 to knockout the TGF-β II receptor in T cells before transduction to make them CAR T cells [142]. Both in vivo and in vitro the edited CAR T cells performed better at eliminating cancer cells through a mechanism of increasing metabolic capacity in glycolysis and oxidative phosphorylation, allowing them to metabolically outcompete cancer cells for resources [142,143]. A similar concept has been applied by others to knock out a variety of genes to improve glycolysis in T cells [144,145,146]. Though this method has shown promising results, it has yet to be translated to the clinic in CAR T-cell trials.

During the expansion phase of CAR T-cell generation, The CAR T cells can be cultured in nutrient restrictive media to mimic the environment the CAR T cells will encounter in the TME. T cells are plastic and can adjust their metabolic preferences depending on their environment during differentiation; thus, restrictive media may improve CAR T-cell longevity and efficacy in the glucose and glutamine-scarce TME [147]. In mouse models, CAR T cells expanded in glutamine and glucose-restrictive media had increased cytotoxic function when injected into the tumor [148,149]. This practice has promise, but characterizing the unique TME metabolic profile of each tumor and customizing the media to match may not be as practical in a clinical application [147].

In a similar vein, CAR T-cell treatment in conjunction with metabolic inhibitors can also improve CAR T-cell efficacy and persistence. PI3K inhibitors block the PI3K/AKT/mTOR pathway, which is regulated strictly in normal cells but heavily relied on for growth in cancer cells [150]. Several PI3K inhibitors have been approved for treatment of aggressive cancers, with other variations in different phases of trial [151]. The combination of PI3K inhibitors with CAR T cells has been shown to improve the reduction of tumor burden in mouse models, likely by reducing the nutrient scarcity burden placed on CAR T cells and improving their persistence [152].

## 5. Conclusions

CAR T cells are an important development in cancer treatment, with their clinical success in hematological malignancies showing much promise. However, solid tumor cancers present obstacles to CAR T cells due to the immunosuppressive TME mediated by pro-tumor cell populations, cytokine profiles, metabolic immunosuppression, vasculature, and more. Though these obstacles are real, research is ongoing to modify CAR T cells or use them in conjunction with other therapies to infiltrate and eliminate tumors more effectively by circumventing immunosuppressive characteristics of the TME (Table 2). This research will continue to expand, enabling obstacles facing CAR T-cell therapy in solid tumors to be overcome in the coming years.

## Figures and Tables

**Table 1:** Common tumor-associated cytokines.

Cytokine/Chemokine	Secreted by	Function
IL-1β	MDSCs TAMs	Mediates the inflammatory response through inflammasomes Induces cancer cell proliferation
IL-2	T cells NK cells	Promotes T-cell growth Influences Treg differentiation Promotes production of TNF-α and INF-γ
IL-4	CD4+ T cells B cells	Influences M2 macrophage polarization Creates a positive feedback loop in CD4+ T cells to produce more IL-4
IL-6	TAMs MDSCs	Promotes inflammation Can lead to accelerated tumor growth by promoting self-renewal in tumor cells
IL-10	TAMs MDSCs Treg cells CD4+ T cells B cells	Influences M2 macrophage polarization Suppresses dendritic cells Negatively regulates the inflammatory response Stimulates T reg cells
IL-12	TAMs Dendritic cells	Promotes differentiation of T cells Enhances production of INF-γ Influences M1 macrophage polarization
IL-13	CD4+ T cells	Influences M2 macrophage polarization
TNF-α	TAMs T cells NK cells	Influences signaling pathways to promote apoptosis or necrosis Promotes cancer cell proliferation
TGF-β	TAMs MDSCs Treg cells CAFs	Dual role in tumor suppression and promotion Inhibits CD8+ T-cell cytotoxic function Reduces MHC presentation in tumor cells Mediates tumor migration and immune cell infiltration
IFN-γ	T cells B cells NK cells Dendritic cells	Dual role in tumor suppression and promotion Activates macrophages Promotes apoptosis Inhibits angiogenesis in the TME

**Table 2:** Overview of immunosuppressive effects on CAR T cells and advancements to overcome them.

Immunosuppressive TME Element	Effect on CAR T Cells	Methods of Overcoming the Limitation
Insufficient vasculature and “shell” surrounding tumors	CAR T-cell homing and tumor infiltration failure	Injection of CAR T cells into tumors, rather than intravenous delivery CAR macrophages
Immunosuppressive cytokine profiles	CAR T-cell exhaustion and anergy	Cytokine secreting CAR T cells and soluble ligand binding T cells
Tumor expression of PDL-1	CAR T-cell exhaustion and death	Checkpoint inhibitor co-treatment and CAR T cells modified to block or alter PD-1/PDL-1 response
High levels of tumor antigen	CAR T-cell exhaustion and anergy	Use of less differentiated T cells in CAR T cells production
Mutation to alter surface expression of neoantigens	Antigen escape	Bispecific and altered binding domain CAR T cells
Hostile metabolic environment	CAR T-cell exhaustion and decreased cytotoxicity	Genetic modification to CAR T-cell’s metabolic processes Expansion phase media modifications (nutrient-limited media, inclusion of metabolic inhibitors)
High levels of immunosuppressive cell populations (Tregs, MDSCs, TAMs)	CAR T-cell anergy	Oncolytic virus co-treatment in conjunction with CAR T cells CAR macrophages
